# Overweight and obesity among women of reproductive age in Mali: what are the determinants?

**DOI:** 10.1093/inthealth/ihaa094

**Published:** 2020-11-18

**Authors:** Abdul-Aziz Seidu, Bright Opoku Ahinkorah, Ebenezer Agbaglo, Albert Apotele Nyaaba

**Affiliations:** Department of Population and Health, University of Cape Coast, Cape Coast, Ghana; College of Public Health, Medical and Veterinary Sciences, James Cook University, Townsville, QLD, Australia; School of Public Health, Faculty of Health, University of Technology Sydney, Sydney, NSW, Australia; Department of English, University of Cape Coast, Cape Coast, Ghana; Department of Population and Health, University of Cape Coast, Cape Coast, Ghana; Youth Harvest Foundation Ghana, Bolgatanga, Ghana

**Keywords:** Mali, obesity, overweight, public health, women

## Abstract

**Background:**

Existing evidence suggests that there has been a surge of overweight and obesity in low- and middle-income countries around the world. In this study we investigated the prevalence and factors associated with overweight and obesity among women in Mali.

**Methods:**

We conducted the study among 5198 women using the 2018 Mali Demographic and Health Survey data. We used binary logistic regression for the analysis and pegged statistical significance at p<0.05.

**Results:**

The prevalence of overweight and obesity was 26.9%. The likelihood of overweight and obesity was high among women 40–44 y of age (adjusted odds ratio [AOR] 5.94 [confidence interval {CI} 4.10 to 8.60]), those who were widowed/divorced/separated (AOR 1.59 [CI 1.04 to 2.43]), those with secondary education (AOR 1.41 [CI 1.13 to 1.75]), richest women (AOR 3.61 [CI 2.63 to 4.95]), those who watched television at least once a week (AOR 1.28 [CI 1.07 to 1.52]) and those who lived in the Kidal region (AOR 10.71 [CI 7.05 to 16.25]). Conversely, the likelihood of overweight and obesity was low among women who belonged to other religions compared with Muslims (AOR 0.63 [CI 0.43 to 0.92]).

**Conclusions:**

This study found a predominance of overweight and obesity among women in Mali. The study showed that age, marital status, education, religion, region of residence, wealth status and frequency of watching television are associated with overweight and obesity among women in Mali. It is therefore critical for public health promotion programs in Mali to sensitize people to the negative effects associated with overweight and obesity. This implies that policies aimed at controlling overweight and obesity in Mali must take these factors into consideration.

## Introduction

Excessive fat accumulation, which could be harmful to the health of humans, is referred to as obesity (or overweight).^1^ Obesity usually occurs when an individual is unable to expend as much energy as he/she takes in,^2^ with the individual developing excessive weight gain.^1^ The past few decades have seen a tremendous increase in cases of obesity, with the majority of the world population living in nation states with high rates of deaths resulting from this health condition.^1^^,^^3^ In the case of women, cases of obesity rose from 69 million in 1975 to 390 million in 2016, which is an increase of about 465%.^4^ This increase in the obesity rate has been attributed to factors such as globalization and socio-economic progress, which have altered the behaviour of humans.^5^

This increase in the obesity rate is critical because of the risks associated with it. For instance, the Global Burden of Disease^6^ has noted that overweight and obesity result in many deaths worldwide. A case in point is that these health conditions killed >3 million people over the world in 2010.^7^ Additionally, there is evidence suggesting that obesity may lead to non-communicable diseases, including 13 different cancers^8^ and hypertension.^9^ Also, obesity exposes women of reproductive age to various health issues, including pre-eclampsia, eclampsia, barrenness and miscarriage.^10^ Obesity is also linked to a decrease in life expectancy among women.^11^

Existing evidence suggests that there has been a surge of overweight and obesity in low- and middle-income countries (LMICs) around the world.^12^ In fact, high rates of obesity have been reported in LMICs such as Bangladesh,^13^ Myanmar,^4^ India,^14^ Nepal,^14^^–^^16^ Ghana^17^^–^^19^ and Ethiopia.^20^ Such studies have revealed some associations between obesity and factors such as living on a highway or a street with no sidewalks,^21^ age, the subjects’ parental obesity,^22^ as well as television watching.^4^^,^^14^^,^^17^^,^^23^ Other studies have shown that frequent watching of television and sedentary behaviours, as well as unhealthy eating habits, often result in weight gain. Television watching also constitutes a health risk with regard to people's exposure to advertisements, which often focus on high-fat and high-sugar foods. These advertisements can influence consumers to adopt an unhealthy diet, ultimately contributing to an increase in their weight.^24^^,^^25^

In the present study we investigate the prevalence and factors associated with overweight and obesity, focusing on women 15–49 y of age in Mali. The present study is critical for several reasons. First, factors associated with overweight and obesity are yet to be assessed in Mali, despite a report that, in 2001, 10% of women in Bamako were obese.^26^^,^^27^ Also, Traore et al.^28^ examined the trends of overweight and obesity among adult women in China, Mali and the USA and found that overweight in Mali has increased from 11.2% to 12.9% between 2002 and 2012—an increase of 1.7%. This increase was greater than that recorded in the USA (1.5%) but the growth was less than that recorded in China (7.2%). Additionally, it has been reported that television watching has increased in Mali, especially among women, because of their interest in watching telenovelas, which have proliferated in recent years.^27^ Furthermore, in Mali there has been a relatively steady increase in urbanisation rate; it increased from 35.2% in 2009 to 43.1% in 2019.^29^

## Methods

### Data source

Data from the 2018 Mali Demographic and Health Survey (MDHS) was used for our analysis. Specifically, the women's recode file was used. The survey is the sixth version since its commencement in 1987.^29^ The MDHS is part of the DHS Program, which aims to monitor health indicators in LMICs. The aim of the survey is to offer reliable estimates of family planning methods, fertility preferences, sexual activity, marriage and other essential population health measures using a two-stage sampling design. At the primary stage, 379 primary survey units (UPSs)/clusters from urban (n=104) and rural (n=275) areas were systematically selected.^29^ These were selected through probability proportional to the size of households from the list of enumeration sections (ESs), which were established during the General Census of Population and Housing (GCPH) conducted in 2009. Household mapping and enumeration in the clusters were organized in order to obtain an updated list of households in each ES. Mapping and enumeration of households were conducted between 25 May and 8 July 2018. However, in the case of the Kidal, Gao and Timbuktu regions, mapping and enumeration were done shortly before the main survey. After this stage, 35 households were sampled from the Kidal, Gao and Timbuktu regions through a systematic selection having equal probability. The same procedure was followed to select 26 households from all the other regions. All women between 15 and 49 y of age who lived in the selected households or were present the night before the survey were considered eligible to participate in the survey. This resulted in a nationally representative sample of 10 519 women. The survey had a 98% response rate for the women.^29^ A detailed description of the methods employed for the survey has been published online at https://dhsprogram.com/what-we-do/survey/survey-display-517.cfm.

### Sample and inclusion criteria

The present study focused mainly on women ages 15–49 y, with the exclusion of pregnant women and those who had given birth within 2 months prior to the data collection.^14^^,^^30^ In all, the sample comprised 5198 women, all of whom had complete data on the variables considered in the study.

### Derivation of variables

#### Outcome variable

Overweight and obesity was the outcome variable of this study. It was generated by dividing the weight by the height squared of each respondent and was expressed as kilograms/meter^2^ (kg/m^2^). Following the World Health Organization's^1^ standard for BMI cut-off points, we generated a binary outcome variable with BMI cut-off points as follows: underweight, <18.5 kg/m^2^; normal weight, 18.5–25 kg/m^2^; overweight, 25.0–29.9 kg/m^2^; and obese, ≥30.0 kg/m^2^. Hence, in order to ensure consistency with prior studies in sub-Saharan Africa (SSA),^17^^–^^19^ for the purpose of this study, a BMI ≥25.0 kg/m^2^ was categorised as overweight and obese and coded as 1, while a BMI <25.0 kg/m^2^ was coded as 0.^17^^–^^19^

#### Independent variables

Eleven independent variables were included in our study. These are age, marital status, level of education, religion, occupation, wealth status, place of residence, region, parity, number of people in the household and frequency of watching television. These variables were selected based on their availability in the dataset and conclusions drawn by some previous studies^4^^,^^14^^,^^17^^–^^19^^,^^23^^,^^30^^,^^31^ on their association with overweight and obesity. In order to make all these variables conceptually meaningful and suitable for the analysis, some of them were recoded. These were marital status, coded as married=0 and cohabiting=1; religion, coded as Islam=0 and other=1; number of people in the household, coded as ≤5=0 and >5=1; and occupation, coded as not working=0 and working=1.

### Statistical analyses

All analyses were conducted in Stata version 14.2 for MacOS (StataCorp, College Station, TX, USA). We first conducted the descriptive weighted analyses, with the aim of determining the sociodemographic characteristics of the study participants (see Table 1). Afterwards, a bivariate analysis was done to determine the differences in the covariates according to the BMI status, using χ^2^, and variables that were significantly associated with overweight and obesity were moved to the multivariable analysis. At that stage we modelled the association between the independent variables and the outcome variable, using binary logistic regression analysis, and the results were reported as adjusted odds ratios (AORs) with their 95% confidence intervals (CIs). We adjusted for age, marital status, level of education, religion, occupation, wealth status, place of residence, region, parity, number of people in the household and frequency of watching television following their availability in the dataset and conclusions drawn by some previous studies^4^^,^^14^^,^^17^^–^^19^^,^^23^^,^^30^^,^^31^ on their association with overweight and obesity. Before the regression analysis, multicollinearity among the covariates was checked using the variance inflation factor (VIF), which showed no evidence of multicollinearity (mean VIF=1.5, maximum VIF=2.1, minimum VIF=1.04). The reference categories for the regress analysis were also informed by previous studies^4^^,^^14^^,^^17^^–^^19^^,^^23^^,^^30^^,^^31^ and a priori. The svy command was applied at this stage in order to correct deficiencies such as under- or overrepresentation.^4^^,^^14^^,^^23^ Weighting was also applied in all the analyses. We followed the Strengthening the Reporting of Observational Studies in Epidemiology statement in conducting this study and writing the manuscript.

**Table 1.** Distribution of overweight and obesity and sociodemographic characteristics of respondents

			Overweight and obesity	
	Weighted	Weighted	No (%)	Yes (%)
Variable	frequency (n)	percentage (%)	73.1	26.9
Age (years) (χ^2^=95.4, p<0.001)				
15–19	1000	19.2	87.2	12.8
20–24	913	17.6	78.7	21.4
25–29	989	19.0	70.4	29.6
30–34	833	16.0	61.9	38.1
35–39	689	13.3	57.6	42.4
40–44	447	8.6	55.8	44.2
45–49	326	6.3	57.1	42.9
Marital status (χ^2^=95.8, p<0.001)				
Never married	791	15.2	82.7	17.3
Married/cohabiting	4257	81.9	68.3	31.7
Widow/divorced/separated	150	2.9	54.0	46.0
Level of education (χ^2^=15.8, p<0.01)				
No education	3473	66.8	71.0	29.0
Primary	691	13.3	70.3	29.7
Secondary	935	18.0	68.6	31.4
Higher	99	1.9	52.7	47.3
Religion (χ^2^=30.6, p<0.001)				
Islam	4877	93.8	69.4	30.6
Other	321	6.2	86.0	14.0
Working status (χ^2^=2.0, p=0.155)				
Not working	2217	42.7	71.1	29.0
Working	2981	57.4	69.3	30.8
Wealth (χ^2^=267.9, p<0.001)				
Poorest	883	17.0	77.3	22.7
Poorer	1055	20.3	81.4	18.6
Middle	995	19.1	78.9	21.1
Richer	1093	21.0	63.0	37.0
Richest	1172	22.6	55.4	44.6
Place of residence (χ^2^=256.9, p<0.001)				
Rural	3851	74.1	77.3	22.7
Urban	1347	25.9	55.7	44.3
Region (χ^2^=337.2, p<0.001)				
Kayes	767	14.8	80.8	19.2
Koulikoro	983	18.9	73.1	26.9
Sikasso	887	17.1	81.1	19.0
Segou	792	15.2	72.5	27.5
Mopti	537	10.3	75.1	24.9
Toumbouctou	197	3.8	65.8	34.2
Gao	147	2.8	79.6	20.5
Kidal	6	0.1	40.5	59.5
Bamako	882	17.0	56.6	43.5
Parity (χ^2^=107.4, p<0.001)				
0	1015	19.5	80.9	19.1
1	656	12.6	75.0	25.0
2	627	12.1	68.5	31.6
3	612	11.8	67.7	32.3
≥4	2288	44.0		
Number of household members (χ^2^=0.5, p=0.468)				
≤5	2054	39.5	69.6	30.4
>5	3144	60.5	70.6	29.5
Frequency of watching television (χ^2^=76.7, p<0.001)				
Not at all	1864	35.9	76.0	24.0
Less than once a week	1156	22.3	71.7	28.3
At least once a week	2178	41.9	63.7	36.3

Source: 2018 Mali Demographic and Health Survey.

## Results

### Bivariate results

Results of the distribution of overweight and obesity across the sociodemographic characteristics of respondents are presented in Table 1. The results indicate that 26.9% of women of reproductive age in Mali were either overweight or obese. The majority of them were 40–49 y of age (44.2%), widow/divorced/separated (46.0%), had higher education (47.3%), were Muslim (30.6%), working (30.8%), richest (44.6%), lived in urban areas (44.3%), resided in the Kidal region (59.5%), had four or more children (32.3%) and had five or fewer household members (30.4%). The results further show that the frequency of watching television (χ^2^=76.7, p<0.001), age (χ^2^=322.6, p<0.001), marital status (χ^2^=95.8, p<0.001), education level (χ^2^=15.8, p<0.01), religion (χ^2^=30.6, p<0.001), wealth status (χ^2^=267.9, p<0.001), place of residence (χ^2^=256.9, p<0.001), region (χ^2^=337.2, p<0.001) and parity (χ^2^=107.4, p<0.001) had statistically significant associations with overweight and obesity among women of reproductive age in Mali.

### Logistic regression analysis results

Table 2 presents results on the factors associated with overweight and obesity in Mali. We found that the likelihood of overweight and obesity was high among women 40–44 y of age (AOR 5.94 [CI 4.10 to 8.60]), those who were widowed/divorced/separated (AOR 1.59 [CI 1.04 to 2.43]), those with secondary education (AOR 1.41 [CI 1.13 to 1.75]), richest (AOR 3.61 [CI 2.63 to 4.95]) and those who lived in the Kidal region (AOR 10.71 [CI 7.05 to 16.25]). Conversely, the likelihood of overweight and obesity was lower among women who belonged to religions other than Muslim (AOR 0.63 [CI 0.43 to 0.92]). We also found that women who watched television at least once a week were more likely to be overweight or obese (AOR 1.28 [CI 1.07 to 1.52]) compared with those who never watched television.

**Table 2.** Binary logistic regression on factors associated with overweight and obesity in Mali

Variables	AOR (95% CI)
Age (years)	
15–19	Ref
20–24	1.50** (1.12 to 2.01)
25–29	2.54*** (1.86 to 3.47)
30–34	4.04*** (2.90 to 5.62)
35–39	4.97*** (3.52 to 7.02)
40–44	5.94*** (4.10 to 8.60)
45–49	5.77*** (3.94 to 8.45)
Marital status	
Never married	Ref
Married/cohabiting	1.58** (1.16 to 2.17)
Widowed/divorced/separated	1.59* (1.04 to 2.43)
Education level	
No education	Ref
Primary	1.25* (1.01 to 1.54)
Secondary	1.41** (1.13 to 1.75)
Higher	1.42 (0.88 to 2.31)
Religion	
Islam	Ref
Other	0.63* (0.43 to 0.92)
Wealth status	
Poorest	Ref
Poorer	1.12 (0.88 to 1.43)
Middle	1.30* (1.02 to 1.65)
Richer	2.88*** (2.26 to 3.67)
Richest	3.61*** (2.63 to 4.95)
Residence	
Rural	Ref
Urban	0.95 (0.74 to 1.23)
Region	
Kayes	Ref
Koulikoro	1.51** (1.14 to 1.99)
Sikasso	1.19 (0.90 to 1.59)
Segou	1.94*** (1.46 to 2.59)
Mopti	1.89*** (1.36 to 2.64)
Toumbouctou	3.37*** (2.51 to 4.52)
Gao	1.25 (0.88 to 1.78)
Kidal	10.71*** (7.05 to 16.25)
Bamako	1.98*** (1.42 to 2.75)
Parity	
0	Ref
1	1.07 (0.79 to 1.45)
2	1.24 (0.89 to 1.71)
3	1.18 (0.85 to 1.66)
4+	1.26 (0.91 to 1.73)
Frequency of watching television	
Not at all	Ref
Less than once a week	1.20 (0.99 to 1.47)
At least once a week	1.28** (1.07 to 1.52)
Pseudo R^2^	0.157
N	5198

Source: 2018 Mali Demographic and Health Survey.

Ref: reference category.

*p<0.05, **p<0.01, ***p<0.001.

## Discussion

The present study investigated the factors associated with overweight and obesity among women 15–49 y of age in Mali. The study revealed that more than a quarter (26.9%) of the sampled women were either overweight or obese. This percentage of obese and overweight women is lower compared with what was recorded in Ghana (40%),^17^ Egypt (85.3%),^32^ Kenya (34.1%)^33^ and Zimbabwe (34.2%),^34^ but higher than what was recorded in Ethiopia (8.8%),^32^ Uganda (16.3%)^35^ and SSA (15.6%).^36^ This difference in the prevalence of obesity recorded in the present study and that reported elsewhere could be attributed to variations in study context as well as differences in socio-economic status. Also, the rate of urbanisation in Mali is relatively low compared with some of these countries.^29^

The study recorded a higher likelihood of overweight and obesity among women who watched television as compared with their counterparts who did not watch television at all. This finding is in agreement with results reported by Gupta et al.,^4^ Piryani et al.,^14^ Tuoriye^17^ and Gupta et al.^23^ in Myanmar, India, Ghana and Nepal, respectively. This association between frequent watching of television and overweight and obesity could be explained within the context of physical inactivity associated with sedentary behaviours such as watching television. In fact, there is literature suggesting that people who often watch television hardly have time to involve themselves in physical activities.^37^ Also, there is evidence that as women spend more time watching television, they are exposed to unhealthy foods that are advertised on television and tend to consume them and become overweight/obese.^23^^,^^25^^,^^38^^,^^39^

Also, the study revealed a significant association between overweight and obesity and age. For instance, we found the likelihood of obesity and overweight to be high among older women. Similar findings were revealed in other sociocultural contexts, such as in Bangladesh^40^^,^^41^ and Ethiopia.^42^ The possible reason for this finding may be that old age is likely to be characterized by high physical inactivity as well as the consumption of more energy-dense foods, which may result in overweight and obesity.^43^ Another possible explanation for this could be that, as people grow, the composition of their body changes, which results in an increase in fat mass and a decline in fat-free mass.^44^^,^^45^

The study also revealed a positive association between socio-economic status (education and wealth) and overweight and obesity. This association is especially evident in the fact that there were regional variations in the likelihood of overweight and obesity in Mali, with a region like Kidal, which is the eighth administrative region of Mali and one of the regions with high poverty levels, having the highest prevalence compared with other regions in the country. This finding is not surprising given that some previous studies noted the association between increased socio-economic status and obesity, especially in LMICs such as Mali,^31^^,^^46^^,^^47^ as opposed to high-income countries.^48^^,^^49^ One of the possible reasons for the finding is that, with advancement in development in LMICs like Mali, people switch from the consumption of healthier staple foods to Western foods, which is likely to result in overweight and obesity.^17^^,^^50^ Another reason for the association between socio-economic status and overweight and obesity is that cultural norms that favour fatter body size constitute one of the drivers of socio-economic differences in overweight and obesity in LMICs, particularly in Africa, and Mali is no exception.^51^ Other studies have explained that women of higher socio-economic status (education and wealth) may have the resources and knowledge of the importance of physical activity and healthy diet but are also faced with several sociocultural barriers that may prevent them from putting these into use.^36^^,^^52^^,^^53^ Such barriers include the belief that higher income and wealth is associated with diets rich in animal fats, which in turn are associated with a higher prevalence of overweight among high socio-economic groups.^36^ People of high socio-economic status may also engage in less physical activity, as their various occupations may give them little or no time to exercise, while others may see physical activity as a hindrance to luxurious living.^52^

We also found an association between religious affiliation and obesity and overweight. Specifically, we found that compared with Muslims, there is less likelihood for women of other religions to be either overweight or obese. This finding can be explained in the context of a study by Kahan^54^ in 38 countries that found high rates of physical inactivity among Muslim women, as well as Benjamin and Donnelly,^55^ who conducted a study on barriers and facilitators influencing the physical activity of Arabic adults. From Benjamin and Donnelly,^55^ the correlates of physical inactivity among Muslim women were tiredness, culturally restrictive sex role and behavioural expectations for women, lack of allocation of funding for women's sports and lack of suitable exercise facilities.^55^ These factors may be linked to the high likelihood of overweight and obesity among Muslim women in Mali. The kinds of foods these women consume could also be influencing their overweight and obesity status. We therefore propose a further longitudinal case–control study and qualitative study to gain a deeper understanding of these findings.

### Strengths and limitations of the study

It is necessary to note some strengths and limitations of this study. First, the two-stage sampling strategy employed, together with the high response rate (98%), reduced selection bias among the women. Additionally, a nationally representative sample was used for this study, which guarantees the generalizability of the findings to all women in Mali. Also, the use of a validated questionnaire and calibrated measuring tools, coupled with highly experienced data collectors, minimized the possibility of measurement errors. Despite these strengths, it is imperative to interpret the findings in the light of some limitations. The first limitation comes with the use of cross-sectional sampling for the study, which makes it virtually impossible to draw causal links between the studied variables. Second, the frequency of television watching was measured in weeks, rather than hours or days.^4^^,^^14^^,^^23^

## Conclusions

The relatively high rate of overweight and obesity reported in the present study presents a public health concern that deserves redress. The study showed that age, marital status, education, religion, region of residence, wealth status and frequency of watching television are associated with overweight and obesity among women in Mali. This suggests that public health policies aimed at reducing overweight and obesity among women of reproductive age in Mali should focus more on the factors that are associated with overweight and obesity. It is necessary for public health promotion programs to create awareness among the Malian population with regard to the negative effects associated with overweight and obesity. This can be done through health education via the various media platforms.

## Data Availability

The dataset can be accessed at https://dhsprogram.com/data/dataset/.

## References

[1] World Health Organization. Obesity and overweight. Available from: http://www.who.int/mediacentre/factsheets/fs311/en/[accessed 21 February 2020].

[2] Romieu I, Dossus L, Barquera S et al. Energy balance and obesity: what are the main drivers? Cancer Causes Control. 2017;28(3):247–58. 28210884 10.1007/s10552-017-0869-z PMC5325830

[3] Ng M, Fleming T, Robinson M et al. Global, regional, and national prevalence of overweight and obesity in children and adults during 1980–2013: a systematic analysis for the Global Burden of Disease Study 2013. Lancet. 2014;384(9945):766–81. 24880830 10.1016/S0140-6736(14)60460-8 PMC4624264

[4] Gupta RD, Sajal IH, Hasan M et al. Frequency of television viewing and association with overweight and obesity among women of the reproductive age group in Myanmar: results from a nationwide cross-sectional survey. BMJ Open. 2019a;9(3):e024680. 10.1136/bmjopen-2018-024680 PMC6475159 30898812

[5] Fox A, Feng W, Asal V. What is driving global obesity trends? Globalization or “modernization”? Global Health. 2019;15(1):32. 31029156 10.1186/s12992-019-0457-y PMC6486955

[6] GBD 2015 Obesity Collaborators. Health effects of overweight and obesity in 195 countries over 25 years. N Engl J Med. 2017;377(1):13–27. 28604169 10.1056/NEJMoa1614362 PMC5477817

[7] Lim SS, Davies MJ, Norman RJ et al. Overweight, obesity and central obesity in women with polycystic ovary syndrome: a systematic review and meta-analysis. Hum Reprod Update. 2012;18(6):618–37. 22767467 10.1093/humupd/dms030

[8] Mokdad AH, Ford ES, Bowman BA et al. Prevalence of obesity, diabetes, and obesity-related health risk factors, 2001. JAMA. 2003;289(1):76–9. 12503980 10.1001/jama.289.1.76

[9] Bâ HO, Camara Y, Menta I et al. Hypertension and associated factors in rural and urban areas Mali: data from the step 2013 survey. Int J Hypertens. 2018;2018:6959165. 29610681 10.1155/2018/6959165 PMC5828104

[10] Sharma R, Biedenharn KR, Fedor JM et al. Lifestyle factors and reproductive health: taking control of your fertility. Reprod Biol Endocrinol. 2013;11(1):66. 23870423 10.1186/1477-7827-11-66 PMC3717046

[11] Olshansky SJ, Passaro DJ, Hershow RC et al. A potential decline in life expectancy in the United States in the 21st century. N Engl J Med. 2005;352(11):1138–45. 15784668 10.1056/NEJMsr043743

[12] World Bank. How tackling the world's deadliest diseases can boost a healthy workforce and economic growth. Available from:https://www.worldbank.org/en/news/immersive-story/2020/02/06/tackling-worlds-deadliest-diseases-can-boost-healthy-workforce-and-economic-growth[accessed 5 November 2020].

[13] Chowdhury MAB, Adnan MM, Hassan MZ. Trends, prevalence and risk factors of overweight and obesity among women of reproductive age in Bangladesh: a pooled analysis of five national cross-sectional surveys. BMJ Open. 2018;8(7):e018468. 10.1136/bmjopen-2017-018468 PMC6059314 30030307

[14] Gupta RD, Haider SS, Sutradhar I et al. Association of frequency of television watching with overweight and obesity among women of reproductive age in India: evidence from a nationally representative study. PLoS One. 2019b;14(8):e0221758. 31465465 10.1371/journal.pone.0221758 PMC6715273

[15] Piryani S, Baral KP, Pradhan B et al. Overweight and its associated risk factors among urban school adolescents in Nepal: a cross-sectional study. BMJ Open. 2016;6(5):e010335. 10.1136/bmjopen-2015-010335 PMC4885275 27207624

[16] Rawal LB, Kanda K, Mahumud RA et al. Prevalence of underweight, overweight and obesity and their associated risk factors in Nepalese adults: data from a nationwide survey, 2016. PLoS One. 2018;13(11):e0205912. 30399189 10.1371/journal.pone.0205912 PMC6219769

[17] Tuoyire DA. Television exposure and overweight and obesity among women in Ghana. BMC Obes. 2018;5:8. 29468075 10.1186/s40608-018-0186-4 PMC5813400

[18] Tuoyire DA, Kumi-Kyereme A, Doku DT. Socio-demographic trends in overweight and obesity among parous and nulliparous women in Ghana. BMC Obes. 2016;3:44. 27826451 10.1186/s40608-016-0124-2 PMC5093993

[19] Doku DT, Neupane S. Double burden of malnutrition: increasing overweight and obesity and stall underweight trends among Ghanaian women. BMC Public Health. 2015;15:670. 26178521 10.1186/s12889-015-2033-6 PMC4502461

[20] Dagne S, Gelaw YA, Abebe Z et al. Factors associated with overweight and obesity among adults in northeast Ethiopia: a cross-sectional study. Diabetes Metab Syndr Obes. 2019;12:391–9. 30962699 10.2147/DMSO.S179699 PMC6434910

[21] Pikora T, Giles-Corti B, Bull F et al. Developing a framework for assessment of the environmental determinants of walking and cycling. Soc Sci Med. 2003;56(8):1693–703. 12639586 10.1016/s0277-9536(02)00163-6

[22] Al-Isa AN. Obesity among Kuwait University students: an explorative study. J R Soc Promot Health. 1999;119(4):223–7. 10673842 10.1177/146642409911900404

[23] Gupta R, Haider SS, Hashan MR et al. Association between the frequency of television watching and overweight and obesity among women of reproductive age in Nepal: analysis of data from the Nepal Demographic and Health Survey 2016. PLoS One. 2020;15(2):e0228862. 32040537 10.1371/journal.pone.0228862 PMC7010261

[24] Bennett GG, Wolin KY, Viswanath K et al. Television viewing and pedometer-determined physical activity among multiethnic residents of low-income housing. Am J Public Health. 2006;96(9):1681–5. 16873736 10.2105/AJPH.2005.080580 PMC1551955

[25] Ghavamzadeh S, Khalkhali HR, Alizadeh M. TV viewing, independent of physical activity and obesogenic foods, increases overweight and obesity in adolescents. J Health Popul Nutr. 2013;31(3):334–42. 24288947 10.3329/jhpn.v31i3.16825 PMC3805883

[26] Diarra M, Sy K, Cissé S. Nutrition et état nutritionnel. Enquête Démographique et de Santé au Mali (EDSM-III). Calverton, MD Macro International Incorporation, Maryland USA. 2001; p. 137–64.

[27] Toure K. *Telenovelas* reception by women in Bouaké (Côte D'Ivoire) and Bamako (Mali). Vis Anthropol. 2007;20(1):41–56.

[28] Traore SS, Amoah AN, Chen H et al. Prevalence of overweight and obesity among adult women in China, Mali and United States: evolution of trends over ten years. Curr Dev Nutr. 2020;4(Suppl 2):1695.

[29] National Institute of Statistics (INSTAT). Planning Statistics Unit Health -Development Sector Social and Family Promotion (CPS/SS-DS-DF) and ICF. 2019. Mali Demographic and Health Survey 2018. Bamako, Mali and Rockville, Maryland, USA: INSTAT, CPS/SS-DS-PF and ICF.

[30] Ntenda PAM, Kazambwe JF. A multilevel analysis of overweight and obesity among non-pregnant women of reproductive age in Malawi: evidence from the 2015–16 Malawi Demographic and Health Survey. Int Health. 2019;11(6):496–506. 30517652 10.1093/inthealth/ihy093

[31] Hashan MR, Gupta RD, Day B et al. Differences in prevalence and associated factors of underweight and overweight and obesity according to rural–urban residence strata among women of reproductive age in Bangladesh: evidence from a cross-sectional national survey. BMJ Open. 2020;10(2):e034321. 10.1136/bmjopen-2019-034321 PMC7045126 32024791

[32] Matos UR, Mesenburg MA, Victora CG. Socioeconomic inequalities in the prevalence of underweight, overweight, and obesity among women aged 20–49 in low-and middle-income countries. Int J Obes. 2020;44(3):609–16. 10.1038/s41366-019-0503-0 PMC7046525 31852998

[33] Mkuu RS, Epnere K, Chowdhury MAB. Prevalence and predictors of overweight and obesity among Kenyan women. Prev Chronic Dis. 2018;15:170401. 10.5888/pcd15.170401 PMC5912924 29679481

[34] Mangemba NT, San Sebastian M. Societal risk factors for overweight and obesity in women in Zimbabwe: a cross-sectional study. BMC Public Health. 2020;20:103. 31992255 10.1186/s12889-020-8215-x PMC6986073

[35] Yaya S, Ghose B. Trend in overweight and obesity among women of reproductive age in Uganda: 1995–2016. Obes Sci Pract. 2019;5(4):312–23. 31452916 10.1002/osp4.351 PMC6700515

[36] Neupane S, Prakash KC, Doku DT. Overweight and obesity among women: analysis of demographic and health survey data from 32 sub-Saharan African countries. BMC Public Health. 2015;16:30. 10.1186/s12889-016-2698-5 PMC4710980 26758204

[37] American Academy of Pediatrics. Policy statement—children, adolescents, obesity, and the media. Pediatrics. 2011;128(1):201–8. 21708800 10.1542/peds.2011-1066

[38] Chen CY, Pereira MA, Kim KH et al. Fifteen-year prospective analysis of television viewing and adiposity in African American and Caucasian men and women: the CARDIA Study. Sage Open. 2015;5(3):2158244015600480.

[39] Subedi YP, Marais D, Newlands D. Where is Nepal in the nutrition transition? Asia Pac J Clin Nutr. 2017;26(2):358–67. 28244717 10.6133/apjcn.112015.10

[40] Sarma H, Saquib N, Hasan MM et al. Determinants of overweight or obesity among ever-married adult women in Bangladesh. BMC Obesity. 2016;3:13. 26962459 10.1186/s40608-016-0093-5 PMC4774107

[41] Abrha S, Shiferaw S, Ahmed KY. Overweight and obesity and its socio-demographic correlates among urban Ethiopian women: evidence from the 2011 EDHS. BMC Public Health. 2016;16:636. 27457223 10.1186/s12889-016-3315-3 PMC4960736

[42] Tanwi TS, Chakrabarty S, Hasanuzzaman S et al. Socioeconomic correlates of overweight and obesity among ever-married urban women in Bangladesh. BMC Public Health. 2019;19:842. 31253123 10.1186/s12889-019-7221-3 PMC6599309

[43] Alemu E, Atnafu A, Yitayal M et al. Prevalence of overweight and/or obesity and associated factors among high school adolescents in Arada sub city, Addis Ababa, Ethiopia. J Nutr Food Sci. 2014;4(2):1000261.

[44] Gallagher D, Visser M, De Meersman RE et al. Appendicular skeletal muscle mass: effects of age, gender, and ethnicity. J Appl Physiol. 1997;83(1):229–39. 9216968 10.1152/jappl.1997.83.1.229

[45] Villareal DT, Apovian CM, Kushner RF et al. Obesity in older adults: technical review and position statement of the American Society for Nutrition and NAASO, The Obesity Society. Obes Res. 2005;13(11):1849–63. 16339115 10.1038/oby.2005.228

[46] Gew CA, Leslie TF, Pawloski LR. Geographic distribution and socio-economic determinants of women's nutritional status in Mali households. Public Health Nutr. 2013;16(9):1575–85. 23072839 10.1017/S136898001200451X PMC10271527

[47] Yaya S, Uthman OA, Ekholuenetale M et al. Socioeconomic inequalities in the risk factors of noncommunicable diseases among women of reproductive age in sub-Saharan Africa: a multi-country analysis of survey data. Front Public Health. 2018;6:307. 30406072 10.3389/fpubh.2018.00307 PMC6207690

[48] Dinsa GD, Goryakin Y, Fumagalli E et al. Obesity and socioeconomic status in developing countries: a systematic review. Obes Rev. 2012;13(11):1067–79. 22764734 10.1111/j.1467-789X.2012.01017.x PMC3798095

[49] McLaren L. Socioeconomic status and obesity. Epidemiol Rev. 2007;29(1):29–48. 17478442 10.1093/epirev/mxm001

[50] Steyn NP, Mchiza ZJ. Obesity and the nutrition transition in sub-Saharan Africa. Ann N Y Acad Sci. 2014;1311(1):88–101. 24725148 10.1111/nyas.12433

[51] Gupta N, Goel N, Shah P et al. Childhood obesity in developing countries: epidemiology, determinants, and prevention. Endocr Rev. 2012;33(1):48–70. 22240243 10.1210/er.2010-0028

[52] Griffiths P, Bentley M. Women of higher socio-economic status are more likely to be overweight in Karnataka, India. Eur J Clin Nutr. 2005;59(10):1217–20. 16077746 10.1038/sj.ejcn.1602228

[53] Subramanian SV, Perkins JM, Özaltin E et al. Weight of nations: a socioeconomic analysis of women in low-to middle-income countries. Am J Clin Nutr. 2011;93(2):413–21. 21068343 10.3945/ajcn.110.004820 PMC3021433

[54] Kahan D. Adult physical inactivity prevalence in the Muslim world: analysis of 38 countries. Prev Med Rep. 2015;2:71–5. 26844051 10.1016/j.pmedr.2014.12.007 PMC4721436

[55] Benjamin K, Donnelly TT. Barriers and facilitators influencing the physical activity of Arabic adults: a literature review. Avicenna. 2013;8:1–8.

